# The prevalence of mental health problems in sub-Saharan adolescents living with HIV: a systematic review

**DOI:** 10.1017/gmh.2020.18

**Published:** 2020-10-26

**Authors:** A.S. Dessauvagie, A. Jörns-Presentati, A.-K. Napp, D.J. Stein, D. Jonker, E. Breet, W. Charles, R. L. Swart, M. Lahti, S. Suliman, R. Jansen, L.L. van den Heuvel, S. Seedat, G. Groen

**Affiliations:** 1 Department of Social Work, Faculty of Business and Social Sciences, Hamburg University of Applied Sciences, Germany; 2 SAMRC Unit on Risk & Resilience in Mental Disorders, Department of Psychiatry and Neuroscience Institute, University of Cape Town, South Africa; 3 Department of Psychiatry and Mental Health, University of Cape Town, South Africa; 4 Faculty of Health and Well-being, Turku University of Applied Sciences, Finland; 5 Department of Psychiatry, Faculty of Medicine and Health Sciences, Stellenbosch University, South Africa; 6 School of Nursing, University of the Free State, South Africa

**Keywords:** Adolescents, epidemiology, Mental health, HIV/AIDS, sub-Saharan Africa

## Abstract

Despite the progress made in HIV treatment and prevention, HIV remains a major cause of adolescent morbidity and mortality in sub-Saharan Africa. As perinatally infected children increasingly survive into adulthood, the quality of life and mental health of this population has increased in importance. This review provides a synthesis of the prevalence of mental health problems in this population and explores associated factors. A systematic database search (Medline, PsycINFO, Scopus) with an additional hand search was conducted. Peer-reviewed studies on adolescents (aged 10–19), published between 2008 and 2019, assessing mental health symptoms or psychiatric disorders, either by standardized questionnaires or by diagnostic interviews, were included. The search identified 1461 articles, of which 301 were eligible for full-text analysis. Fourteen of these, concerning HIV-positive adolescents, met the inclusion criteria and were critically appraised. Mental health problems were highly prevalent among this group, with around 25% scoring positive for any psychiatric disorder and 30–50% showing emotional or behavioral difficulties or significant psychological distress. Associated factors found by regression analysis were older age, not being in school, impaired family functioning, HIV-related stigma and bullying, and poverty. Social support and parental competence were protective factors. Mental health problems among HIV-positive adolescents are highly prevalent and should be addressed as part of regular HIV care.

## Introduction

In sub-Saharan Africa, HIV is a major cause of adolescent morbidity and mortality. Of the 1.6 million adolescents living with HIV globally, around 1.1 million reside in Eastern and Southern Africa and another 430 000 in West and Central Africa (UNICEF, 2019). Approximately 35 000 adolescents died of HIV in both regions, and nearly 190 000, the majority of them adolescent girls, got newly infected in 2017 (UNICEF, 2018a; 2018b). HIV is associated with adolescent mental health problems in both, high- and low-income settings (Mellins and Malee, 2013; Vreeman *et al*., 2017), with social exclusion and HIV-related stigma playing an important role (Boyes *et al*., 2019). Mental health problems among HIV-positive adolescents have been linked to poor adherence to antiretroviral treatment (ART) and a higher risk of substance abuse and sexual risk behaviors, leading to less favorable health outcomes and a higher risk of HIV transmission (Mellins and Malee, 2013; Dow *et al*., 2016; Vreeman *et al*., 2017).

In the African context, data on adolescent mental health is scarce and capacities for mental health care are limited, as is the case in many low-income settings (Fisher and Cabral de Mello, 2011; Erskine *et al*., 2017; WHO, 2018; UNICEF, 2018c). Regarding the mental health of HIV-positive adolescents in sub-Saharan Africa, numerous studies have been published in recent years (Kamau *et al*., 2012; Louw *et al*., 2016; Lwidiko *et al*., 2018; Hoare *et al*., 2019; West *et al*., 2019). To our knowledge, there is no recent review which specifically summarizes epidemiological data from different sub-Saharan settings and reports on the quality of these studies. A recent review by Vreeman *et al*. (2017) included studies from both high-income and low-income settings and included a broad age range (aged < 10 and up to 24). Another review on mental health problems of perinatally infected HIV-positive youth predominantly included studies from the United States (Mellins and Malee, 2013). With our review, we sought to close this gap by summarizing the existing evidence on the prevalence of mental health problems among HIV-positive adolescents (aged 10–19) in sub-Saharan Africa. Additionally, we explored associated sociodemographic, health-related, and community factors, as documented in the included studies.

## Methods

This study formed part of an overarching systematic review that explores the prevalence of mental health problems in general adolescent populations in sub-Saharan Africa as well as in risk groups (HIV/AIDS, poverty, or exposure to trauma). The systematic review aims at updating the findings from the review of Cortina *et al*. (2012) on child mental health in sub-Saharan Africa that included studies up to 2008. It was registered with the PROSPERO International prospective register of systematic reviews at the National Institute for Health Research (PROSPERO 2018 CRD42018112853) and will be published in separate subsections. Due to the number of retrieved studies, we decided to publish the results of the systematic review in subsections, with this section focusing on the prevalence of mental health problems specifically among HIV-positive adolescents, who constitute one of our a priori risk groups.

The systematic review was undertaken by the MEGA project team. MEGA is an international collaborative project for mental health promotion among adolescents in South Africa and Zambia (Lahti *et al*., 2020; MEGA 2020). The project aims to build capacity for adolescent mental health among health care workers in primary care settings by training the trainers in higher education institutions in both countries.

### Search strategy

An extensive database search was conducted in PubMed, Scopus, and PsycINFO in June and November 2018, covering a 10-year period. Additional studies were retrieved from Google Scholar, from reference lists and citations of the included studies or through contact with other researchers. A second search was conducted in January 2020 to include articles that were published since our search in 2018. Only peer-reviewed studies reporting prevalence data and published in English were included.

The COCOPOP scheme was used to define inclusion and exclusion criteria for the database search (Joanna Briggs Institute, 2017)

− *Context:* sub-Saharan Africa, defined according to the World Bank Country and Lending Groups (World Bank, n.d.).
− *Condition:* mental health problems or clinical diagnoses, as assessed by standardized questionnaires or diagnostic interviews.
− *Population:* adolescents aged 10–19, residing in sub-Saharan Africa.

Exclusion criteria were:

− publications on populations with a broader age range than 10–19 that do not report separate prevalence data for adolescents between 10 and 19;
− psychiatric clinical populations (publications on clinical populations from HIV care were included);
− lack of prevalence data;
− non-standardized or incomplete instruments, not regularly used in mental health research;
− reviews, validation studies, or qualitative studies.

The following search terms were used: child*, youth, adolesc*; sub-Saharan, Africa, South Africa, Zambia; prevalence, incidence, epidemiol*; psychiat*, mental, depress*, ADHD, anxiety (see supplementary material, Table 2). The database search revealed 1374 articles. In total, 65 additional articles were found through Google Scholar, further 22 through reference lists, citations, and contact with other researchers. After the removal of duplicates, 1070 records were left for the screening of title and abstract. After exclusion of articles on clinical psychiatric populations or youth beyond the age range of 10–19 and articles with a wrong publication type or date, 301 articles were eligible for full-text assessment.

**Fig. 1.**
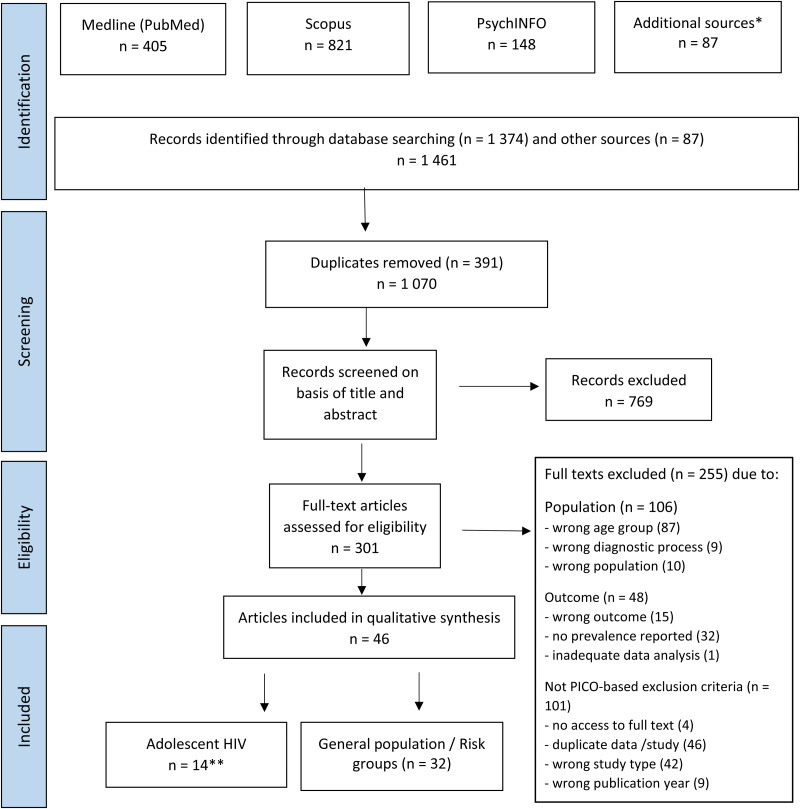
PRISMA Flow-chart. *65 articles found from Google Scholar, 22 from reference lists, citations or author contact. **found from PubMed (1), PsycINFO (4), Scopus (3), Google Scholar (6), through reference lists (2), recommendation by other researchers (3); four articles were found in more than one source.

The exclusion of articles was done according to the PICO-based taxonomy (Edinger and Cohen, 2013). All articles were independently evaluated by two researchers. Fourteen articles focusing on the prevalence of mental health problems among HIV-positive adolescents were included in this sub-review (Fig. 1). Six articles regarding HIV-affected adolescents or adolescents from high-prevalence communities (antenatal HIV-prevalence >30%) were included in the second systematic review (to be published separately; see supplementary material, Table 3).

### Data extraction and analysis

The following scheme was used for data extraction: publication year, land/region, study design, sample origin, sampling method, sample size, distribution males/females, age range, risk factors, instruments, informant, data collection process, prevalence of psychological symptoms or mental disorder, and additional findings. Due to the broad heterogeneity of instruments and cut-offs used to assess mental health problems across studies, a meta-analysis was not performed. Instead, data were analyzed and presented in a descriptive, narrative overview. Results from statistical analyses of associated factors were also included in the analysis. Only factors reaching a significance level of *p* = 0.05 or less were considered as being significantly associated.

Quality assessment of the studies was based on the Joanna Briggs Institute (JBI) Critical Appraisal Checklist for Studies Reporting Prevalence Data (Munn *et al*., 2015). This was extended to cultural appropriateness (see supplementary material, Table 4).

## Results

Fourteen studies from eight different countries were included. All of the studies except one were conducted in Eastern and Southern Africa, most of them in countries with a high burden of the HIV epidemic. Study designs were mainly cross-sectional, there were two case-control studies and one mixed-methods study of which only the quantitative findings are reported here (see supplementary material, Table 4). In eight of the studies, screening scales were used to assess mental health symptoms or behavior (Table 2). Four studies reported a disorder prevalence, assessed by either diagnostic interviews or symptom count score. Two studies (Musisi and Kinyanda, 2009; Woollett *et al*., 2017) used both. Because Woollett *et al*., indicated that cut-offs were set to identify symptomatic adolescents and not used for diagnostic purposes, the results are reported in the section on symptom prevalence. Most of the studies assessed point prevalence. Exceptions are marked in Table 4, supplementary material.

Sample sizes ranged between 82 and 1339 (Table 2). For four of the studies, only a subsample could be included in the review (Menon *et al*., 2009; Ng *et al*., 2015; Bankole *et al*., 2017; Kinyanda *et al*., 2019). Most samples included older (15–19) and younger (10–14) adolescents. Exceptions were the study by Vreeman *et al*., and Menon *et al*., who focused on younger adolescents and Okawa *et al*., who focused on older adolescents (Menon *et al*., 2009; Vreeman *et al*., 2015; Okawa *et al*., 2018). Females and males were almost equally distributed, with females being slightly overrepresented (58–62%) in five of the studies. The characteristics of the study samples are shown in Table 1.

**Table 1.** Sociodemographic and health-related factors of the study participants

Study	Age range (years) and gender	Social environment	HIV-related health status and treatment
Ashaba et al. (2018)	13-17 m:f = 41:59	22% orphans, 65% with HIV-positive caregiver 27% lived with both parents	All on ART (mean duration 8.5 yrs.) All knew their HIV status
Bankole et al. (2017)	8-16 (only ALHIV aged 11-16 included) m:f = 41:59	^b^32% single orphans, 22.7% double orphans 17.3% with academic failure	^b^89% on HAART 64% with advanced clinical disease^a^
Gentz *et al*. (2017)	12-18 m:f = 48:52	37.4% had both parents alive 17.2% lost both parents 32.3% reported one or more days without enough food per week 49.5% lived in informal housing	All on ART (mean duration 7 yrs.) All knew their HIV status 3.9% with advanced immunosuppression^a^
Kemigisha *et al*. (2019)	10-19 m:f = 38:62	36.6% single orphans, 15.5% double orphans 82.4% primary school students 0.9% without formal education 15.2% lived in temporary housing	95.2% on ART (median duration 5 years) 3.4% with advanced clinical disease^a^
Kim *et al*. (2015)	12-18 m:f = 44:56	19.9% had both parents as primary caregivers 31.3% not enrolled in school or primary school not completed	93.6% on ART 81.3% knew their HIV status 71.3% with advanced clinical disease^a^ 31.9% with advanced immunosuppression^a^
Kinyanda *et al*. (2019)	5-17 (only ALHIV aged 12-17 included) m:f = 48:52	42% single orphans 19.8% double orphans 20.2% lived with both parents	94.6% on ART 16.5% with advanced immunosuppression^a^
Lyambai and Mwape (2018)	11-17 m:f = 42:58	63.5% lived with a single parent 25% lived with other relatives 11% lived with both parents	All on ART treatment 64% unaware of their HIV status
Menon *et al*. (2009)	11-15 m:f = 46:54	26.8% had their mother as a primary caregiver	73.2% were on ART 58.3% did not know their HIV status 42.5% reported health problems
Musisi and Kinyanda (2009)	10-18 m:f = 44:56	53.7% double orphans 2.4% had both parents alive 75,6% enrolled in school	All knew their HIV status 60.9% with advanced clinical disease^a^
Ng *et al*. (2015)	10-17 m:f = 49:51	^b^52% raised by a single caregiver 29% orphaned 90% were attending school	Not given
Okawa *et al*. (2018)	15-19 m:f = 42:58	64.7% orphans, 24.7% double orphans 17.9% did not complete basic education	94.2% on ART (mean duration 6 yrs.)
Smith Fawzi *et al*. (2016)	10-17 m:f = 50:50	29% with mother as head of the household 29% with father as head of the household 93% attending school, 74% with lack of basic necessities during the past 6 months	All on ART
Vreeman *et al*. (2015)	10-14 m:f = 46:54	50% lost both parents 99% were enrolled at school 34% had difficulties in attending	All on HAART (mean duration 4,4 yrs.) 43% knew their HIV status 37% with advanced clinical disease^a^
Woollett *et al*. (2017)	13-19 m:f = 46:54	73% orphaned 98% attending school	88% on ART 89% knew their HIV status

^a^advanced immunosuppression = CD4 count < 350/mm^3^, advanced clinical disease = WHO stage 3 or 4

^b^applies to the whole study group

**Table 2.** Overview of the studies included

	Setting	Sample size	Instruments and informant/s	Mental health outcome
*Assessment of psychiatric disorder with diagnostic interview or symptom count score*				
Ashaba *et al*. (2018)	South-Western Uganda HIV clinic attached to Mbarara Regional Referral Hospital and University of Science and Technology	*n* = 224	MINI International Neuropsychiatric Interview for children and adolescents^a^	Major depressive disorder: 16% Suicidality (past month): 14% (4% low, 6% medium, 4% high risk)
Bankole *et al*. (2017)	South Nigeria Pediatric Outpatient Clinic of the University of Calabar Teaching Hospital (UCTH)	*n* = 150 (75 ALHIV, 75 controls; only 31 ALHIV included)	MINI International Neuropsychiatric Interview for children and adolescents^a^	Depressive disorder: 41.9%
Kim *et al*. (2015)	Malawi two large HIV clinics: one in South-Eastern Malawi, one in Lilongwe	*n* = 562	Children´s Depression Rating Scale, Revised (CDRS-R)^a^	Depressive disorder: 18.9%
Kinyanda *et al*. (2019)	Uganda 5 HIV clinics in Central and South-Western Uganda	*n* = 1 339 (patients and caregivers; only 479 ALHIV included)	Child and Adolescent Symptom Inventory-5 (CASI-5)^b^, caregiver-reported Youth´s Inventory-4R (YI-4R)^b^, self-reported	Any psychiatric disorder: 23.8% Any behavioral disorder: 12.4% Any emotional disorder: 18.2% Major depressive disorder: 5.2% Any anxiety disorder: 14.7% ADHD: 6.4% (either caregiver- or self-report)
*Assessment of psychological symptoms or behavior with screening scales*				
Gentz *et al*. (2017)	Namibia Pediatric ARV Clinic of a State Hospital in Katutura, Windhoek	*n* = 99	SDQ, self-reported^b^	Emotional and behavioral difficulties (past 6 months): 12.2% scored in the abnormal range, 16.3% in the borderline range
Kemigisha *et al*. (2019)	South-Western Uganda Mbarara Municipal Council Health Center IV and Mbarara Regional Referral Hospital (MRRH)	*n* = 336	Center for Epidemiologic Studies Depression Scale for Children (CES-DC)^a^	Depressive symptoms: 45.8% (42.5% in males; 47.8% in females) Age 10–14: 37.4% Age 15–19: 62.3% Suicidal ideation (past 6 months): 7.7%
Lyambai and Mwape (2018)	Zambia Choma General Hospital, a second level referring hospital	*n* = 103	SDQ, self- and parent-reported^a^	Emotional and behavioral difficulties (past 6 months): 29.2% (self-reported) *v.* 34.3% (parent-reported) scored in the abnormal range
Menon *et al*. (2009)	Zambia University Teaching Hospital Lusaka 5 other clinics in the district 5 basic schools (grade 5–9)	*n* = 547 (127 ALHIV, 420 controls; only ALHIV included)	SDQ, self-reported^a^	Emotional and behavioral difficulties (past 6 months): 29.1% (ALHIV) *v.* 27.8% (controls) scored in the abnormal and borderline range
Ng *et al*. (2015)	Rwanda catchment area of two district hospitals, rural Rwanda	*n* = 683 (218 ALHIV, 228 adolescents with an HIV-positive caregiver, 237 unaffected adolescents; only ALHIV included)	Youth Self-Report (YSR), internalizing subscale^b^	Suicidal ideation and behavior (6 months): 21.1% in ALHIV Suicidal behavior (6 months): 21% in HIV-positive and HIV-affected adolescents *v.* 13% in unaffected adolescents
Okawa *et al*. (2018)	Zambia University Teaching Hospital in Lusaka	*n* = 190	Center for Epidemiologic Studies Depression Scale (CES-D)^a^, short form	Depressive symptoms: 25.3%
Smith Fawzi *et al*. (2016)	Rwanda catchment area of two district hospitals, rural Rwanda	*n* = 193 (subsample from Ng *et al*.)	Center for Epidemiologic Studies Depression Scale for Children (CES-DC)^b^	Depressive symptoms: 26%
Vreeman *et al*. (2015)	Western Kenya 8 healthcare facilities for HIV treatment	*n* = 285	SDQ, self-reported^a^ Patient Health Questionnaire (PHQ-9)^a^	Emotional and behavioral difficulties (past 6 months): 5% scored in the abnormal range and 4% in the borderline range Depressive symptoms: 19% (15% minimal, 3% minor, 2% moderate/severe symptoms)
*Combined use of screening scales and diagnostic interviews*				
Musisi and Kinyanda (2009)	Uganda Child and Adolescent Clinic, specialized in HIV care, in Kampala	*n* = 82	SRQ-25^a^ psychiatric interview	Significant psychological distress (past 30 days): 51.2% Depressive disorder: 40.8% Anxiety disorder: 45.6% Somatization: 18% Suicide attempts (12 months): 17.1% Suicide attempts (lifetime): 19.5%
Woollett *et al*. (2017)	South Africa 5 pediatric ARV clinics serving adolescents in Johannesburg	*n* = 343	Child Depression Inventory (CDI)^a^, short version Revised Children´s Manifest Anxiety Scale (RCMAS)^a^ Child PTSD Checklist^a^ MINI International Neuropsychiatric Interview^a^, suicidality subscale	Symptomatic for Depression: 14% Anxiety: 25% PTSD: 5% Symptomatic for any disorder: 27% Suicidal ideation (past month): 24% Suicidal behavior (past month): 5%

ALHIV = adolescents living with HIV

a instrument previously used or validated in other African settings (Chipimo and Fylkesnes, 2010; Chishinga *et al*., 2011; Betancourt *et al*., 2012; Boyes *et al*., 2012; Boyes and Cluver, 2013; Cholera *et al*., 2014; Kim *et al*., 2014),

b pilot study conducted or local validation of instruments (Betancourt *et al*., 2012; Mpango *et al*., 2017)

### Prevalence of psychological symptoms

Four studies reported *emotional and behavioral difficulties,* assessed by the Strengths and Difficulties Questionnaire (SDQ) (Menon *et al*., 2009; Vreeman *et al*., 2015; Gentz *et al*., 2017; Lyambai and Mwape, 2018). The lowest prevalence was reported by Vreeman *et al*., from a sample of younger adolescents (*n* = 285; age 10–14) enrolled in a disclosure intervention trial in Kenya (Vreeman *et al*., 2015): 9% scored in the borderline range and 5% in the clinical range. Two other studies, conducted in Zambia (*n* = 99; age 12–18) and Namibia (*n* = 127; age 11–15), reported a prevalence of 28.5% and 29.1% for emotional and behavioral problems (borderline and clinical range) (Menon *et al*., 2009; Gentz *et al*., 2017). Another study from Zambia (*n* = 103; age 11–17) reported on the percentage that scored in the clinical range (29.2%) (Lyambai and Mwape, 2018). Menon *et al*., compared a sample of HIV-positive adolescents (*n* = 127) to a sample of school children (*n* = 420) (Menon *et al*., 2009) and found a comparable prevalence of emotional and behavioral problems (29.1 *v.* 27.8%). Three studies also reported results for the different subscales of the SDQ (Menon *et al*., 2009; Gentz *et al*., 2017; Lyambai and Mwape, 2018): In the sample from Namibia (Gentz *et al*., 2017), emotional problems were more prevalent (22%) than conduct problems (12.2%), peer problems (10.9%) or hyperactivity/inattention (4%). The two other studies, both from Zambia, found a higher frequency of peer problems (46.9% and 41.8%, respectively), compared to emotional or conduct problems (Menon *et al*., 2009; Lyambai and Mwape, 2018). Peer problems were also frequent in the sample of unaffected school children that was used as a control group (34.4%). Lyambai and Mwape compared self-rated results to parent-rated results and found that parents reported fewer problems in each of the categories (Lyambai and Mwape, 2018) and this was most prominent for peer problems.

Musisi and Kinyanda (2009) made use of the Self-Reporting Questionnaire 25 (SRQ-25) to assess *significant psychological distress* among a sample of HIV-positive adolescents (*n* = 82; age 10–18) from Uganda and reported a prevalence of 51%.

*Symptoms of depression* were assessed in five of the studies (Vreeman *et al*., 2015; Smith Fawzi *et al*., 2016; Woollett *et al*., 2017; Okawa *et al*., 2018; Kemigisha *et al*., 2019). In a sample from Zambia (*n* = 190; age 15–19), 25.3% of adolescents had high scores of depressive symptoms, according to the short form of the Center for Epidemiologic Studies Depression Scale (CES-D) (Okawa *et al*., 2018). Of the total, 69% of symptomatic adolescents were female and 31% were male. A similar prevalence (26%) was reported in a study from Rwanda (*n* = 193; age 10–17) that used the Center for Epidemiologic Studies Depression Scale for Children (CES-DC) (Smith Fawzi *et al*., 2016). In a younger sample from western Kenya (*n* = 285; age 10–14), 19% of adolescents scored positive for depression, according to the Patient Health Questionnaire (PHQ-9). However, 15% showed minimal, 3% minor, and 2% moderate or severe symptoms (Vreeman *et al*., 2015). In a sample from deprived urban neighborhoods of Johannesburg, South Africa, 14% of adolescents (*n* = 343; median age 16) showed symptoms of depression, according to the Child Depression Inventory (CDI) (Woollett *et al*., 2017)

*Anxiety and posttraumatic stress disorder (PTSD)* were only reported from the South African sample (Woollett *et al*., 2017). Assessment with the Revised Children´s Manifest Anxiety Scale (RCMAS) and the Child PTSD Checklist revealed that 25% of adolescents had symptoms of anxiety and 5% had symptoms of PTSD. Furthermore, 27% of participants were symptomatic for either depression, anxiety, or PTSD. Female adolescents had significantly higher scores of depression, anxiety, or PTSD, compared to male adolescents.

Three studies assessed *suicidality:* In the sample of Woollett *et al*., 24% reported suicidal ideation and 5% suicide attempts during the previous month. Ng *et al*. (2015) reported on suicidal ideation and behavior from Rwanda, using the Youth Self-Report (YSR), internalizing subscale. In their matched case-control study (*n* = 683; age 10–17), 21% of HIV-positive adolescents reported suicidal behavior (including self-harm) during the previous 6 months, compared to 13% of unaffected adolescents. Kemigisha *et al*. (2019) reported a lower prevalence of suicidal ideation (7.7%) from a sample in western Uganda (*n* = 336; age 10–19). In total, 69.2% of adolescents reporting suicidal ideation were female and 30.8% were male. Furthermore, 81% of adolescents reporting suicidality also scored positive for depression.

### Prevalence of mental disorders

The prevalence of mental disorders was assessed by structured or semi-structured diagnostic interviews [MINI International Neuropsychiatric Interview, Children´s Depression Rating Scale-Revised (CDRS-R)] in three studies (Kim *et al*., 2015; Bankole *et al*., 2017; Ashaba *et al*., 2018). Another study used symptom count scores from the Child and Adolescent Symptom Inventory-5 (CASI-5) and the Youth´s Inventory-4R (YI-4R) (Kinyanda *et al*., 2019). Musisi and Kinyanda (2009) assessed mental disorders in an ICD-10-based, diagnostic psychiatric interview, not further specified.

An overall prevalence of *any psychiatric disorder* was reported from a sample of perinatally infected adolescents in Uganda (*n* = 479; age 12–17) (Kinyanda *et al*., 2019). Based on symptom count scores (either caregiver- or self-report), 23.8% of adolescents scored positive for a psychiatric disorder, with 18.2% scoring positive for any *emotional disorder* and 12.4% for any *behavioral disorder.* The level of comorbidity between emotional and behavioral disorders was 38.6% and 22.5%, respectively. ADHD was the most prevalent behavioral disorder (6.4%) and anxiety disorders were the most prevalent type of emotional disorders (14.7%). The prevalence of major depressive disorder was 5.2%. Prevalence according to self-report alone were much lower (ADHD 3.1%, anxiety 10.1%, major depressive disorder 0.2%).

The prevalence of *depressive disorder* was 18.9% in a large sample (*n* = 562; age 12–18) from Malawi (Kim *et al*., 2015). A comparable prevalence (16%) was found in a sample from rural Uganda (*n* = 224; age 13–17) (Ashaba et al., 2018) and 14% of the sample reported suicidality in the previous month, with 4% having a high suicide risk.

In a sample from Nigeria (*n* = 31; age 11–16), 41.9% were diagnosed with *depression,* with the highest prevalence (83.3%) found in the 14–16 years age group (Bankole *et al*., 2017). For their whole study group (aged 6–16), Bankole *et al*., showed that *depression and suicidality* were more prevalent among ALHIV, compared to controls. An older study from Uganda (*n* = 82; age 10–18) found a *depression* prevalence of 40.8% (Musisi and Kinyanda, 2009). In the same sample, 45.6% of adolescents were diagnosed with an *anxiety disorder,* 18% with *somatization disorder,* and 1.2% with *bipolar disorder (mania).* Furthermore, 19.5% reported ever having made a *suicide attempt* and 17.1% had attempted suicide within the past 12 months.

### Associations with sociodemographic, health-related and community factors

Twelve studies assessed correlations between mental health and explanatory factors, either by chi-square or t-test (Table 3) or by regression analysis (Table 4).

**Table 3.** Mental health outcomes and correlations found by bivariate analysis

	Mental health variable	Positive correlation with	Negative correlation with	No significant correlation
Ashaba (2018)	Major depressive disorder	Stigma, bullying, duration on ART		Age, gender, orphanhood, HIV status of the caregiver
	Suicidality	Stigma, bullying		Age, gender, orphanhood, HIV status of the caregiver, duration on ART
Bankole (2017)	Depression	Older age, academic failure, living with relatives, orphanhood, more than one hospitalization		Gender, WHO clinical stage, CD4 count, HAART
Gentz (2017)	Emotional and behavioral difficulties (SDQ)	Days without food, missed ARV doses, having disclosed HIV status to others, stigma, negative self-image, public attitude	Support through caregiver and friends, child-specific assets	HIV-related health variables (CD4 count, viral load, time on ART), age of disclosure, time since disclosure
Kemigisha (2019)	Depression	Older age, time to clinic > 30 min, sexual risk behavior, having disclosed HIV status to others	Taking taxi/motorcycle for transport to the clinic	Gender, parental death, WHO clinical stage, adherence
Kim (2015)	Depression	Fewer years of schooling, change in caregiver, experience of forced sex or abuse, being bullied, dissatisfaction with physical appearance, older age at disclosure, worse immunological state, being malnourished	Mother/father as caregiver	Family income, location of home, time to clinic, failed school term, hospital admissions (past year), being on ART, history of TB treatment, WHO clinical stage
Kinyanda (2019)	Emotional disorders	Older age, higher caregiver education		Gender, urban/rural study site, type of caregiver, orphanhood, education level, SES, CD4 count, being on ART
	Behavioral disorders	Older age, male gender		Urban/rural study site, type of caregiver, orphanhood, education level, SES, CD4 count, being on ART, caregiver education
Lyambai and Mwape (2018)	Number of mental health difficulties (SDQ)	Higher level of stigma		
Menon (2009)	Emotional and behavioral difficulties (SDQ)	Self-reported health problems		Taking ARV
Ng (2015)	Suicidal ideation and behavior	Older age, single caregiver, not being in school, child depression, child conduct problems, caregiver mental health problems, severe physical punishment, child-reported HIV-stigma	Good parenting, community support	Gender
	Suicidal behavior only	Positive HIV status, HIV status of the caregiver	Higher SES	
Musisi and Kinyanda (2009)	Psychological distress	Older age, being out of school, not being able to play sports at school, HIV status of the caregiver		Academic performance
Okawa (2018)	Depression	Experience of HIV-associated stigma	Good relationship with family, friends, health care workers	Age, gender, education level, parental survival, basic HIV knowledge
Woollett (2017)	Depression, anxiety or PTSD	Days hungry, peer violence, having been inappropriately touched, hit or attacked, female gender, feeling to belong	Knowledge of HIV status, feeling able to control the future, having dreams for the future, having a safe place in the community	Age, looking after younger children or sick family members, lost someone close, not talked about loss, having been hospitalized, having disclosed HIV status, time since the disclosure

**Table 4.** Sociodemographic, family, and community factors associated with mental health found by multiple logistic or hierarchical regression

	Associated with	Study	Type and strength of correlation (p-value)
***Sociodemographic factors***			
Older age	Depression	Kim (2015)	Positive (0.004)
	Emotional and behavioral disorder	Kinyanda (2019)	Positive (< 0.01 and 0.007)
Female gender	Suicidality	Ashaba (2018)	Positive (0.03)
Male gender	Behavioral disorder	Kinyanda (2019)	Positive (0.01)
Child assets	Total difficulties (SDQ)	Gentz (2017)	Negative (0.027)
Not in school	Depression	Kim (2015)	Positive (0.005)
Higher caregiver education	Emotional disorder	Kinyanda (2019)	Positive (0.03)
***HIV-related factors***			
Duration on ART	Depression	Ashaba (2018)	Positive (0.02)
Travel time to clinic (> 30 min)	Depression	Kemigisha (2019)	Positive (0.041)
Unsatisfactory relationship with health worker	Depression	Okawa (2018)	Positive (< 0.01)
***Family factors***			
Good parenting	Suicidal ideation and behavior	Ng (2015)	Negative (0.03; 0.001)
Child mental health symptoms	Suicidal ideation and behavior	Ng (2015)	Positive (0.001; 0.03)
Caregiver mental health symptoms	Suicidal ideation	Ng (2015)	Positive (0.03)
Unsatisfactory relationship with family	Depression	Okawa (2018)	Positive (< 0.01)
***Community factors***			
Bullying	Depression	Ashaba (2018)	Positive (0.04)
	Suicidality	Ashaba (2018)	Positive (0.02)
Being bullied for taking medications	Depression	Kim (2015)	Positive (< 0.0001)
Stigma	Total difficulties (SDQ)	Gentz (2017)	Positive (0.011)
	Emotional symptoms (SDQ)	Gentz (2017)	Positive (0.02)
	Suicidality	Ashaba (2018)	Positive (0.02)
	Depression	Okawa (2018)	Positive (0.01)

Findings on associated factors from bivariate analyses were often contradictory (Table 3). This applied to most of the sociodemographic variables (age, gender, socioeconomic status, education level/academic performance, orphanhood, HIV status of the caregiver). For health-related variables, WHO clinical stage, CD4 count, or being on ART were not associated with mental health problems in most studies, while self-reported health problems, being out of school, and not being able to play sports in school were. Disclosure of HIV or time since the disclosure was not associated with mental health problems, whereas two studies found an association between having disclosed HIV status to others and poor mental health. Findings on mental health and ART adherence were also contradictory.

Bullying and stigma were consistently associated with poor mental health outcomes. Poverty (days hungry or non-availability of child assets) and the experience of violence or abuse were associated with poor mental health. Social support through community, family, or friends, and good parenting were associated with better mental health outcomes in several studies. Individual-level factors such as feeling able to control the future and having dreams for the future were likewise associated with better mental health.

Factors identified by logistic regression are shown in Table 4. Apart from sociodemographic and HIV-related factors, family factors (good parenting, relationship with family, child and caregiver mental health) were identified as important predictors of mental health. With regard to community-level factors, bullying and stigma predicted poor mental health outcomes.

Two other studies explored the factors associated with non-adherence, using mental health as an independent variable in logistic regression (Smith Fawzi *et al*., 2016; Okawa *et al*., 2018). Smith Fawzi *et al*., found a significant association between conduct problems and non-adherence and also, though weaker, between self-reported depression and non-adherence. Okawa *et al*., did not find a significant association between depressive symptoms and non-adherence.

## Discussion

The vast majority of adolescents living with HIV reside in sub-Saharan Africa. To date, there has not been a specific review of the burden of mental health problems for this high-risk population in this region of the world. We summarized the relevant evidence for this high-risk group as part of a systematic review on mental health problems among sub-Saharan adolescents based on peer-reviewed studies published between 2008 and 2019. Collectively, the studies indicated a high prevalence of mental health problems, with 24–27% of adolescents scoring positive for any psychiatric disorder and 30–50% showing emotional or behavioral difficulties or significant psychological distress. Based on regression analyses, older age, not being in school, poverty, and bullying and stigma predicted mental health problems. Social support and parental competence were protective.

The high prevalence of mental health problems among HIV-positive adolescents found in this review aligns with previous research on HIV-positive adolescents in both, high- and low-income settings (Mellins and Malee, 2013; Vreeman *et al*., 2017). The prevalence of emotional and behavioral problems, depression, and anxiety was in the same range as the prevalence reported by Mellins and Malee from the USA, while ADHD was much more common in the US studies. Case-control studies indicated a higher prevalence of suicidality and depression among HIV-positive adolescents, compared to controls (Ng *et al*., 2015; Bankole *et al*., 2017), while the prevalence of emotional and behavioral problems did not differ between the two groups (Menon *et al*., 2009).

### Associations with sociodemographic, health-related, and community factors

Sociodemographic factors associated with mental health problems in regression analyses were older age, poverty, not being in school, and higher caregiver education. Unsatisfactory relationships with health workers, longer travel time to clinic, and duration on ART were health-related factors associated with poor mental health. Stigma and bullying were strong community-level predictors for mental health problems. Factors associated with better mental health outcomes included social support and good parenting.

The factors described above are not much different from the risk factors known for mental health problems in general adolescent populations (Patel *et al*., 2007; Fisher and Cabral de Mello, 2011; Kieling *et al*., 2011; WHO, 2012; WHO, 2013a).This raises the question of whether it is the HIV infection itself or rather environmental and family factors that pose a risk to mental health (Mellins and Malee, 2013; Vreeman *et al*., 2017). As the majority of HIV-positive adolescents in sub-Saharan Africa were perinatally infected, they also have to cope with the consequences of familial AIDS: bereavement, caring for ill family members with AIDS, stigma and discrimination, poverty, lack of social support and guidance and diminished educational opportunities (Lowenthal *et al*., 2014). A study from Kenya on vertically infected and HIV-affected adolescents found similar depression scores in both groups, with orphanhood, poverty, and caregiver depression being associated factors (Abubakar *et al*., 2017). Ng *et al*. (2015) found similar rates of suicidality among HIV-positive and HIV-affected adolescents and correlations with caregiver´s mental health. The relevance of caregiver health and child-caregiver relationship for mental health outcomes in this population is known from previous research (Bhana *et al*., 2016; Louw *et al*., 2016; Boyes *et al*., 2019).

### HIV disclosure and adherence to ART

The disclosure of HIV was not identified as a predictive factor for mental health in regression models. The bivariate analysis suggested no adverse effects of disclosure, but associations between mental health problems and disclosure of HIV status to others. WHO strongly recommends timely disclosure (WHO, 2013b). Studies from this review found that knowledge of HIV status was associated with better mental health (Woollett *et al*., 2017), while older age at disclosure was associated with mental health problems (Kim *et al*., 2015). Ramos *et al*. showed that HIV-positive youth (aged 11–24) who had to figure out their HIV status on their own were more likely to show mental health symptoms and internal stigma, compared to youth who were disclosed to (Ramos *et al*., 2018). Incomplete adherence to ART was also more likely among youth not disclosed to.

Findings on mental health as an independent factor for ART adherence were contradictory (Smith Fawzi *et al*., 2016; Okawa *et al*., 2018). Other studies reported a positive association between poor mental health and non-adherence (Dow *et al*., 2016; Vreeman *et al*., 2017) or virologic failure (Lowenthal *et al*., 2012). A systematic review of the factors associated with adherence to ART in LMIC did not identify mental health as one of the most prominent factors for adherence (Hudelson and Cluver, 2015). A large study from the Eastern Cape, South Africa, found that perinatally infected adolescents were more likely to be adherent, compared to behaviorally infected adolescents (Sherr *et al*., 2018). Simultaneously, behaviorally infected adolescents showed higher scores of depression, anxiety, and suicidality and were more likely to report internalized stigma and substance use. None of the studies included in this review explored the mode of infection as a predictive factor for mental health and/or adherence. The study of Sherr *et al*., suggests that the mode of infection might be an important factor for both mental health outcomes and retention in care and that it also has an influence on how adolescents are treated by health care workers.

### Implications for HIV care

Given the high prevalence of mental health problems among HIV-positive adolescents, identifying and addressing these problems is crucial. Screening for mental health problems and integrating mental health care into regular HIV services is highly recommended (Musisi and Kinyanda, 2009; Kim *et al*., 2015; Bankole *et al*., 2017; Gentz *et al*., 2017; Woollett *et al*., 2017; Lyambai and Mwape, 2018; Okawa *et al*., 2018).

Lyambai and Mwape (2018) conducted qualitative interviews among nurses working at an ART clinic. Mental health literacy among health care workers was low and there was no dedicated mental health service for HIV-positive adolescents available. HIV-positive adolescents face multiple challenges in the context of HIV: daily adherence to medications, coping with the diagnosis, coping with an AIDS-ill caregiver and/or bereavement, coping with stigma and discrimination from peers, disclosure to potential partners, and negotiating safer sex (Lowenthal *et al*., 2014; Bryant and Beard, 2016). For many adolescents, the transition from pediatric services to adult HIV care is critical, with a high risk of discontinuation of treatment at this point (Lowenthal *et al*., 2014; Bryant and Beard, 2016; Vreeman *et al*., 2017). Addressing their psychosocial needs and well-being is crucial to keeping adolescents in care.

There are multiple approaches for improving the mental health of HIV-positive adolescents, e.g. enhancement of self-regulation skills and coping strategies (Bhana *et al*., 2016; Mutumba *et al*., 2017), and strengthening resources for social support (Casale *et al*., 2019). As HIV likely affects the whole family, there is a need for evidence-based family interventions, the VUKA family program being one promising example (Bhana *et al*., 2014; Mellins *et al*., 2014). Addressing stigma is another important issue. To inform mental health promotion and program planning, it is crucial to understand the psychosocial challenges HIV-positive adolescents face (Petersen *et al*., 2010; Ashaba *et al*., 2019a).

### Differences in methodology and prevalence between the studies

As different samples are exposed to a different set of risk and protective factors, differences in prevalence are comprehensible (Fisher *et al*., 2011; Kieling *et al*., 2011). Apart from community and family factors, the percentage of adolescents receiving ART, differences in HIV-related physical health, and the quality of HIV care will have an impact on the prevalence of mental health problems (Okawa *et al*., 2018; Boyes *et al*., 2019).

Two studies on younger adolescents reported a low prevalence of emotional and behavioral problems, almost comparable to the general adolescent population (Menon *et al*., 2009; Vreeman *et al*., 2015). This may be due to the fact that the prevalence of mental health problems among children and adolescents rises with age (de Girolamo *et al*., 2012; WHO, 2017).

Differences between self- and caregiver-report were described in two of the studies (Lyambai and Mwape, 2018; Kinyanda *et al*., 2019). van den Heuvel *et al*. (2019) explored agreement and discrepancies between caregiver- and self-reported results from the sample of Kinyanda *et al*., and only found a modest correlation between the two. A low inter-informant agreement was also reported by Doku and Minnis (2016) from a sample of HIV-affected children and their caregivers. Thus, the prevalence of mental health problems may vary according to the type of informant.

Reviews on child and adolescent mental health found that studies that used screening instruments reported higher rates of mental health problems, compared to studies that used diagnostic interviews (Fisher *et al*., 2011; Cortina *et al*., 2012). This is also true for most of the studies in this review. Only two studies with very small sample sizes that employed diagnostic interviews reported exceptionally high rates of depressive disorder (Musisi and Kinyanda, 2009; Bankole *et al*., 2017).

### Limitations

Most of the studies used self-reporting screening instruments. As screening instruments can merely identify symptomatic people or people with a probable psychiatric disorder, results from screening instruments are not equivalent to disorder prevalence. Using screening instruments can result in an overestimation of disorder prevalence, as could be shown for depression screening among people living with HIV in sub-Saharan Africa (Tsai, 2014). Particularly when used in settings with a low expected prevalence, there is a considerable risk of misclassification (Kagee et al., 2013; Stockings *et al*., 2015).

Self-reported results are prone to reporting bias and may be influenced by social desirability, so results have to be interpreted with caution. Where sensitive issues are concerned, there is a considerable risk of underreporting. Most studies used convenience sampling which has an impact on the representativeness of the data. As the vast majority of studies were cross-sectional, no causal relationships can be derived from the results. Most of the studies did not use control groups, which makes it difficult to differentiate between HIV-related mental health risks and risks that adolescents share with their peers from the same community.

The majority of standardized screening instruments and diagnostic interviews in use in the field of child and adolescent mental health today were developed in high-income settings. Questions developed and tested in high-income settings may be inappropriate when used in a low-resource setting, which can lead to an over- or underestimation of prevalence (Sweetland *et al*., 2014; Owen *et al*., 2016). For many of the standardized instruments used today, there are no clinical cut-offs validated for Africa (de Vries *et al*., 2018; Hoosen *et al*., 2018). Only a few screening instruments were either developed with HIV-positive or HIV-affected adolescents in sub-Saharan Africa or were validated and adapted for use within this population (Betancourt *et al*., 2011; Ng *et al*., 2014; Mutumba *et al*., 2015; Ashaba *et al*., 2019b). The majority of these instruments have not been used on a larger scale. The inclusion of studies that used locally developed instruments and also of qualitative studies could have led to a more precise understanding of the mental health issues in the HIV-positive adolescent population. To achieve better comparability of the data, we focused on prevalence rates that were determined using standardized measures only. By only including studies reporting point prevalence data, we cannot draw any conclusions on the trajectories of adolescents living with HIV. This is a crucial topic for future research. For children orphaned by AIDS, Cluver *et al*. (2012) have shown that mental health problems worsened over time. This may also be true for HIV-positive adolescents.

The number of databases that were searched was limited due to time and capacity restrictions, though the most important ones were included. Because we focused on peer-reviewed articles only, EMBASE and conference websites were not searched. The inclusion of studies in other languages than English may have led to additional findings. Because of our focus on adolescents aged 10–19, publications employing a broader age than 10–19, reporting prevalence data for children and adolescents or for adolescents and young people up to the age of 24 may have been missed. Due to publication bias, studies that found a high prevalence of mental health problems may be overrepresented. As the review was conducted within an adolescent mental health promotion project in South Africa and Zambia, both countries were included in the search terms which might have led to an overrepresentation of studies from these two countries. Furthermore, the results presented here are a subsection of a larger systematic review and the comparability of the findings to the general sub-Saharan adolescent population, as well as other high-risk groups, can only be commented on once the other sections of the review have been published. Lastly, the review was limited to the period 2008 and 2019 and so studies published before and after this period, which may be informative, were excluded.

## Conclusion

This review updates and synthesizes evidence on the prevalence of mental health problems among HIV-positive adolescent populations in sub-Saharan Africa. Mental health problems are highly prevalent in this population and need to be addressed within regular HIV care settings. Poor mental health can be associated with non-adherence to ART and with other risk behaviors, leading to poorer physical outcomes and a higher risk of HIV transmission. Health care professionals working with HIV-positive adolescents should be enabled to recognize mental health problems and respond to them in an appropriate, non-discriminatory way to ensure the best possible outcomes.

## Supplementary material

For supplementary material accompanying this paper visit http://dx.doi.org/10.1017/gmh.2020.18. click here to view supplementary material
