# Supplementary material for: The prevalence of mental health problems in sub-Saharan adolescents living with HIV: a systematic review
Source: Glob Ment Health (Camb). 2020 Oct 26;7:e29. doi: 10.1017/gmh.2020.18 (PMC7786273; doi:10.1017/gmh.2020.18)
Supplement: Supplementary file 1 [file S2054425120000187sup001.pdf]

Online material, Table 1: Glossary.

Adolescents (WHO)	People aged 10 to 19, with the differentiation between younger adolescents (10 to 14) and older adolescents (15 to 19)
Young people (WHO)	People aged 10 to 24
Youth (UN, WHO)	People aged 15 to 24
Sub-Saharan Africa (World Bank Country and Lending Groups)	Angola, Benin, Botswana, Burkina Faso, Burundi, Cabo Verde, Cameroon, Central African Republic, Chad, Comoros, Democratic Republic of Congo, Republic of Congo, Cote d´Ivoire, Equatorial Guinea, Eritrea, Ethiopia, Gabon, Gambia, Ghana, Guinea, Guinea-Bissau, Kenya, Lesotho, Liberia, Madagascar, Malawi, Mali, Mauretania, Mauritius, Mozambique, Namibia, Niger, Nigeria, Rwanda, Sao Tomé and Principe, Senegal, Seychelles, Sierra Leone, Somalia, South Africa, South Sudan, Sudan, Swaziland, Tanzania, Togo, Uganda, Zambia, Zimbabwe
African Region (WHO)	all Sub-Saharan countries apart from Somalia and Sudan; contrary to Sub-Saharan Africa, the WHO African Region includes Algeria
ALHIV	Adolescents living with HIV
People living with HIV	HIV-positive people; the term implies the fact that a person may live and be productive many more years, even if diagnosed with HIV
People affected by HIV	includes family members and others who are involved in the caregiving of a person living with HIV, often used for children and partners
AIDS orphans	children who have lost one or both parents because of HIV. UNAIDS suggests not to use this term, but to use “orphans and other children made vulnerable by AIDS” instead
HIV clinical stage (WHO)	Progression of HIV from stage 1 (asymptomatic or generalized lymphadenopathy) to stage 4 (severe weight loss/malnutrition, AIDS-defining infections, HIV encephalopathy)
CD4 count	Marker for the HIV-induced immunosuppression, from none/non-significant (> 500/mm^3) to advanced/severe (< 350/mm^3)
Definitions are predominantly based on the UNAIDS Terminology Guidelines 2015 (https://www.unaids.org/sites/default/files/media_asset/2015_terminology_guidelines_en.pdf) and on definitions from WHO (https://www.who.int/hiv/pub/guidelines/arv2013/intro/keyterms/en/).	

Online material, Table 2: Search strategy

Database	Full search term
PubMed Medline	(child*[Title/Abstract] OR youth*[Title/Abstract] OR adolesc*[Title/Abstract])) AND ("south africa"[Title/Abstract] OR zambia[Title/Abstract] OR sub-saharan[Title/Abstract] OR africa[Title/Abstract])) AND (prevalence[Title/Abstract] OR incidence[Title/Abstract] OR epidemiol*[Title/Abstract])) AND (psychiat*[Title/Abstract] OR mental[Title/Abstract] OR depress*[Title/Abstract] OR ADHD[Title/Abstract] OR anxiety[Title/Abstract])<br>Filter: publication date: 10 years
Scopus	TITLE-ABS ((child* OR youth* OR adolesc*) AND ("south africa" OR zambia OR sub-saharan OR subsaharan OR africa) AND (prevalence OR incidence OR epidemiol*) (psychiat* OR mental OR depress* OR adhd OR anxiety)) AND (LIMIT-TO (PUBYEAR , 2008, 2009, 2010, 2011, 2012, 2013, 2014, 2015, 2016, 2017, 2018, 2019))
PsycINFO (assessed by Ovid)	((adolesc* or child* or youth*) and ("south africa" or zambia or sub-saharan or africa) and (prevalence or incidence or epidemiol*) and (psychiat* or mental or depress* or ADHD or anxiety))<br>+ limit to abstracts<br>+ Publication year 2008 - 2019

Online material, Table 3: Full-text HIV articles not included, with reasons for exclusion. *articles on HIV-affected adolescents and adolescents orphaned by AIDS

**Population**	
Wrong age group	Barenbaum E, Smith T (2016).* Social support as a protective factor for children impacted by HIV/AIDS across varying living environments in southern Africa. AIDS Care 28 Suppl 2, 92–99.
	Bhana A, Mellins CA, Small L, Nestadt DF, Leu C-S, Petersen I, Machanyangwa S, McKay M (2016). Resilience in perinatal HIV+ adolescents in South Africa. AIDS Care 28 Suppl 2, 49–59.
	Brittain K, Myer L, Phillips N, Cluver LD, Zar HJ, Stein DJ, Hoare J (2019). Behavioural health risks during early adolescence among perinatally HIV-infected South African adolescents and same-age, HIV-uninfected peers. AIDS Care 31, 131–140.
	Dow DE, Turner EL, Shayo AM, Mmbaga B, Cunningham CK, O'Donnell K (2016). Evaluating mental health difficulties and associated outcomes among HIV-positive adolescents in Tanzania. AIDS Care 28, 825–833.
	Kamau JW, Kuria W, Mathai M, Atwoli L, Kangethe R (2012). Psychiatric morbidity among HIV-infected children and adolescents in a resource-poor Kenyan urban community. AIDS Care 24, 836–842.
	Louw K-A, Ipser J, Phillips N, Hoare J (2016). Correlates of emotional and behavioural problems in children with perinatally acquired HIV in Cape Town, South Africa. AIDS Care 28, 842–850.
	Lowenthal E, Lawler K, Harari N, Moamogwe L, Masunge J, Masedi M, Matome B, Seloilwe E, Gross R (2012). Rapid psychosocial function screening test identified treatment failure in HIV+ African youth. AIDS Care 24, 722–727.
	Lwidiko A, Kibusi SM, Nyundo A, Mpondo BCT (2018). Association between HIV status and depressive symptoms among children and adolescents in the Southern Highlands Zone, Tanzania: A case-control study. PLoS ONE 13, e0193145.
	Ramos JV, Mmbaga BT, Turner EL, Rugalabamu LL, Luhanga S, Cunningham CK, Dow, DE (2018). Modality of primary HIV disclosure and association with mental health, stigma, and antiretroviral therapy adherence in Tanzanian youth living with HIV. AIDS Patient Care and STDs 32, 31–37.
	West N, Schwartz S, Mudavanhu M, Hanrahan C, France H, Nel J, Mutunga L, Bernhardt S, Bassett J, Van Rie A (2019). Mental health in South African adolescents living with HIV. AIDS Care 31, 117–124.
	Zeegers I, Rabie H, Swanevelder S, Edson C, Cotton M, van Toorn R (2010). Attention deficit hyperactivity and oppositional defiance disorder in HIV-infected South African children. Journal of Tropical Pediatrics 56, 97–102.
Wrong population	Mellins CA, Elkington KS, Leu CS, Santamaria EK (2012). Prevalence and change in psychiatric disorders among perinatally HIV-infected and HIV-exposed youth. AIDS Care 24, 953–62.
	Mellins CA, Malee KM (2013). Understanding the mental health of youth living with perinatal HIV infection: lessons learned and current challenges. Journal of the International AIDS Society 16, 18593.
	Shiferaw G, Bacha L, Tsegaye D (2018). Prevalence of depression and its associated factors among orphan children in orphanages in Ilu Abba Bor Zone, South West Ethiopia. Psychiatry Journal 2018, 6865085.
**Outcome**	
Wrong outcome	Meinck F, Cluver LD, Boyes ME (2015). Household illness, poverty and physical and emotional child abuse victimisation: findings from South Africa's first prospective cohort study. BMC Public Health 15, 444.
No prevalence reported	Abubakar A, Van de Vijver FJR, Hassan AS, Fischer R, Nyongesa MK, Kabunda B, Berkley JA, Stein A, Newton CR (2017). Cumulative psychosocial risk is a salient predictor of depressive symptoms among vertically HIV-infected and HIV-affected adolescents at the Kenyan coast. Annals of Global Health 83, 743–752.
	Betancourt T, Scorza P, Kanyanganzi F, Fawzi MCS, Sezibera V, Cyamatare F, Beardslee W, Stulac S, Bizimana JI, Stevenson A, Kayiteshonga Y (2014). HIV and child mental health: a case-control study in Rwanda. Pediatrics 134, e464-472.
	Boyes ME, Bowes L, Cluver LD, Ward CL, Badcock NA (2014).* Bullying victimisation,

	internalising symptoms, and conduct problems in South African children and adolescents: a longitudinal investigation. Journa of Abnormal Child Psychology 42, 1313–1324.
	Boyes ME, Cluver LD (2015).* Relationships between familial HIV/AIDS and symptoms of anxiety and depression: the mediating effect of bullying victimization in a prospective sample of South African children and adolescents. Journal of Youth and Adolescence 44, 847–859.
	Boyes ME, Cluver LD, Meinck F, Casale M, Newnham E (2019). Mental health in South African adolescents living with HIV: correlates of internalising and externalising symptoms. AIDS Care 31, 95–104.
	Casale M, Boyes M, Pantelic M, Toska E, Cluver L (2019). Suicidal thoughts and behaviour among South African adolescents living with HIV: Can social support buffer the impact of stigma? Journal of Affective Disorders 245, 82–90.
	Casale M, Cluver L, Crankshaw T, Kuo C, Lachman JM, Wild LG (2015).* Direct and Indirect Effects of Caregiver Social Support on Adolescent Psychological Outcomes in Two South African AIDS-Affected Communities. American Journal of Community Psychology 55, 336–346.
	Cluver L, Gardner F, Operario D (2009).* Poverty and psychological health among AIDS-orphaned children in Cape Town, South Africa. AIDS Care 21, 732–741.
	Cluver LD, Gardner F, Operario D (2008).* Effects of stigma on the mental health of adolescents orphaned by AIDS. Journal of Adolescent Health 42, 410–417.
	Cluver LD, Orkin M, Gardner F, Boyes ME (2012).* Persisting mental health problems among AIDS-orphaned children in South Africa. J Child Psychol Psychiatry 53, 363–370.
	Govender K, Reardon C, Quinlan T, George G (2014). Children's psychosocial wellbeing in the context of HIV/AIDS and poverty: a comparative investigation of orphaned and non-orphaned children living in South Africa. BMC Public Health 14, 615.
	Mutumba M, Bauermeister JA, Harper GW, Musiime V, Lepkowski J, Resnicow K, Snow RC (2017). Psychological distress among Ugandan adolescents living with HIV: Examining stressors and the buffering role of general and religious coping strategies. Global Public Health 12, 1479–1491.
	Nduwimana E, Mukunzi S, Ng LC, Kirk CM, Bizimana JI, Betancourt TS (2017). Mental health of children living in foster families in rural Rwanda: The role of HIV and the family environment. AIDS and Behavior 21, 1518–1529.
	Onuoha FN, Munakata T (2010). Inverse association of natural mentoring relationship with distress mental health in children orphaned by AIDS. BMC Psychiatry 10, 6.
	Reardon C, Gavin G, Mucheuki C, Govender K, Quinlan T (2015). Psychosocial and health risk outcomes among orphans and non-orphans in mixed households in KwaZulu-Natal, South Africa. African Journal of AIDS Research 14, 323–331.
	Sharer M, Cluver L, Shields JJ, Ahearn F (2016).* The power of siblings and caregivers: under-explored types of social support among children affected by HIV and AIDS. AIDS Care 28 Suppl 2, 110–117.
	Sherr L, Cluver LD, Toska E, He E (2018). Differing psychological vulnerabilities among behaviourally and perinatally HIV infected adolescents in South Africa - implications for targeted health service provision. AIDS Care 30, 92–101.
**Not PICO-based exclusion criteria**	
No full-text access	Olang'o CO, Nyamongo IK, Nyambedha EO (2012).* Children as caregivers of older relatives living with HIV and AIDS in Nyang'oma division of western Kenya. African Journal of AIDS Research 11, 135–142.
Duplicate study/data	Barhafumwa B, Dietrich J, Closson K, Samji H, Cescon A, Nkala B, Davis J, Hogg RS, Kaida A, Gray G, Miller CL (2016).* High prevalence of depression symptomology among adolescents in Soweto, South Africa associated with being female and cofactors relating to HIV transmission. Vulnerable Children and Youth Studies 11, 263–273.
	Mpango RS, Kinyanda E, Rukundo GZ, Levin J, Gadow KD, Patel V (2017). Prevalence and correlates for ADHD and relation with social and academic functioning among children and adolescents with HIV/AIDS in Uganda. BMC Psychiatry 17, 336.
	van den Heuvel LL, Levin J, Mpango RS, Gadow KD, Patel V, Nachega JB, Seedat S, Kinyanda E (2019). Agreement and discrepancy on emotional and behavioral problems between caregivers and HIV-infected children and adolescents from Uganda. Frontiers in

	Psychiatry 10, 460.
Wrong study type	Ashaba S, Cooper-Vince C, Vořechovská D, Maling S, Rukundo GZ, Akena D, Tsai AC (2019). Development and validation of a 20-item screening scale to detect major depressive disorder among adolescents with HIV in rural Uganda: A mixed-methods study. SSM Population Health 7, 100332. (validation study)
	Ashaba S, Cooper-Vince CE, Vořechovská D, Rukundo GZ, Maling S, Akena D, Tsai AC (2019b). Community beliefs, HIV stigma, and depression among adolescents living with HIV in rural Uganda. Afr J AIDS Res 18, 169–180. (qualitative study)
	Binagwaho A, Fawzi MCS, Agbonyitor M, Nsanzimana S, Karema C, Remera E, Mutabazi V, Shyirambere C, Cyamatare P, Nutt C, Wagner C, Condo J, Misago N, Kayiteshonga Y (2016). Validating the Children's Depression Inventory in the context of Rwanda. BMC Pediatrics 16, 29. (validation study)
	Kim MH, Mazenga AC, Devandra A, Ahmed S, Kazembe PN, Yu X, Nguyen C, Sharp C (2014). Prevalence of depression and validation of the Beck Depression Inventory-II and the Children's Depression Inventory-Short amongst HIV-positive adolescents in Malawi. Journal of the International AIDS Society 17, 18965. (validation study)
	Mpango, R.S., Kinyanda, E., Rukundo, G.Z., Gadow, K.D., Patel, V., 2017. Cross-cultural adaptation of the Child and Adolescent Symptom Inventory-5 (CASI-5) for use in central and south-western Uganda: the CHAKA project. Tropical Doctor 47, 347–354. (validation study)
	Mutumba M, Resnicow K, Bauermeister JA, Harper GW, Musiime V, Snow RC, Lepkowski JM (2015). Development of a psychosocial distress measure for Ugandan adolescents living with HIV. AIDS and Behavior 19, 380–392. (validation study)
	Ng LC, Kanyanganzi F, Munyanah M, Mushashi C, Betancourt TS (2014). Developing and Validating the Youth Conduct Problems Scale-Rwanda: A Mixed Methods Approach. PLoS One 9. (validation study)
	Petersen I, Bhana A, Myeza N, Alicea S, John S, Holst H, McKay M, Mellins C (2010). Psychosocial challenges and protective influences for socio-emotional coping of HIV+ adolescents in South Africa: a qualitative investigation. AIDS Care 22, 970–978. (qualitative study)
	Skovdal M, Ogutu VO (2009).* "I washed and fed my mother before going to school": understanding the psychosocial well-being of children providing chronic care for adults affected by HIV/AIDS in western Kenya. Global Health 5, 8. (qualitative study)
**Publications on HIV-affected adolescents and adolescents orphaned by AIDS (included, but published separartely)**	
Closson K, Dietrich JJ, Nkala B, Musuku A, Cui Z, Chia J, Gray G, Lachowsky NJ, Hogg RS, Miller CL, Kaida A (2016).* Prevalence, type, and correlates of trauma exposure among adolescent men and women in Soweto, South Africa: implications for HIV prevention. BMC Public Health 16, 1191.	
Cluver L, Orkin M (2009).* Cumulative risk and AIDS-orphanhood: interactions of stigma, bullying and poverty on child mental health in South Africa. Social Science and Medicine 69, 1186–1193.	
Cluver L, Orkin M, Boyes ME, Sherr L, Makasi D, Nikelo J (2013).* Pathways from parental AIDS to child psychological, educational and sexual risk: developing an empirically-based interactive theoretical model. Social Science and Medicine 87, 185–193.	
Doku PN, Minnis H (2016).* Multi-informant perspective on psychological distress among Ghanaian orphans and vulnerable children within the context of HIV/AIDS. Psychological Medicine 46, 2329–2336.	
Lachman JM, Cluver LD, Boyes ME, Kuo C, Casale M (2014).* Positive parenting for positive parents: HIV/AIDS, poverty, caregiver depression, child behavior, and parenting in South Africa. AIDS Care 26, 304–313.	
Thurman TR, Nice J, Taylor TM, Luckett B (2017).* Mitigating depression among orphaned and vulnerable adolescents: a randomized controlled trial of interpersonal psychotherapy for groups in South Africa. Child and Adolescent Mental Health 22, 224–231.	

Online material, Table 4: Quality assessment

Study	Sample frame	Recruitment	Sample size	Description of the study sample	Coverage of sample	Valid instruments	Measurement	Statistical analysis	Response rate	Comments	Overall appraisal
Ashaba et al. (2018)	Y	Y	Y (224)	Y	U	Y	Y	Y	U	consecutive sampling in HIV clinic, sample size calculated, no response rate reported, all measures translated to local language	good
Bankole et al. (2017)	Y	Y	Y (150)	Y	U	Y	Y	Y	U	case-control study, consecutive sampling in HIV clinic, age- and sex-matched HIV-negative controls from the same hospital; no response rate reported, only subsample (n = 31) included in the analysis	good
Gentz et al. (2017)	Y	Y	Y (99)	Y	Y	Y	Y	Y	Y	convenience sampling in ARV clinic, sample size calculation, 20 pilot interviews conducted	good
Kemigisha et al. (2019)	Y	Y	Y (336)	Y	Y	Y	Y	Y	Y	convenience sampling in urban and rural HIV clinics, interviews in a local language or English, instrument validated in the region	very good
Kim et al. (2015)	Y	Y	Y (562)	Y	Y	Y	Y	Y	Y	convenience sampling in 2 large HIV clinics, instruments validated in Malawi by the author	very good
Kinyanda et al. (2019)	Y	Y	Y (1339)	Y	Y	Y	Y	Y	Y	convenience sampling, recruitment in 5 HIV clinics, sample size calculation, instruments translated and locally adapted only subsample (n = 479) included in the analysis	very good
Lyambai & Mwape (2018)	Y	Y	Y (103)	Y	U	Y	Y	Y	U	mixed-methods study, systematic random sampling, small sample size, translation and assistance for participants with low literacy	moderate
Menon et al. (2009)	Y	U	Y (547)	Y	Y	Y	Y	Y	U	case-control study, convenience sampling in HIV clinics (n = 127) and schools (n = 420), low response rate in the school sample (59.8%), 89% in clinical sample	moderate
Musisi & Kinyanda (2009)	Y	Y	Y (82)	Y	U	Y	Y	Y	U	purposive sampling in HIV clinic, sample size calculation, small sample size, no response rate reported	moderate
Ng et al. (2015)	Y	Y	Y (683)	Y	Y	Y	Y	Y	Y	case-control study, stratified multi-step random sampling in the catchment area of 2 district hospitals, controls not matched for age and gender, sample size calculation, all measures locally validated with 378 child-caregiver dyads, Rwandan research assistants, local languages used	very good

Okawa et al. (2018)	Y	Y	Y (190)	Y	Y	Y	Y	Y	Y	convenient sampling in a university teaching hospital, instrument validated with adults in Zambia, assistance for participants with low literacy	very good
Smith Fawzi et al. (2016)	Y	Y	Y (193)	Y	Y	Y	Y	Y	Y	cross-sectional analysis of a subsample from Ng et al.	very good
Vreeman et al. (2015)	Y	Y	Y (285)	Y	U	Y	Y	Y	U	convenience sampling in 8 healthcare facilities, only adolescents aged 10-14 included, no response rate reported, questionnaires in a local language or English (baseline data from a disclosure intervention trial)	good
Woollett et al. (2017)	Y	Y	Y (343)	Y	U	Y	Y	Y	U	convenience sampling in 5 pediatric ARV clinics, no response rate reported, 50% cut-off used because a validated clinical cut-off is missing	good

Y = yes, U = unclear
All studies used a cross-sectional design unless otherwise stated. For a detailed description of the quality categories see: Checklist for Prevalence Studies, Joanna Briggs Institute, 2017
